# Disparities in healthcare access experienced by Hispanic chronic kidney disease patients: a cross-sectional analysis

**DOI:** 10.1186/s41043-024-00508-4

**Published:** 2024-01-31

**Authors:** Sadia Anjum Ashrafi, Rifat Binte Alam, Alicia Kraay, Babatope Ayokunle Ogunjesa, Andiara Schwingel

**Affiliations:** Department of Kinesiology and Community Health, University of Illinois Urbana-Champaign, Champaign, IL USA

**Keywords:** Chronic kidney disease, Hispanics, Healthcare access, Equity, Racial disparities

## Abstract

**Background:**

Chronic kidney disease (CKD) is a public health concern, and the disease disproportionately affects Hispanics. Improved healthcare access for Hispanic CKD patients can reduce the disease burden. This study assesses the healthcare access disparities experienced by Hispanic CKD patients compared to Whites.

**Methods:**

We analyzed three National Health and Nutrition Examination Survey (NHANES) datasets for 2013–2014, 2015–2016, and 2017–2018. The primary predictor variable was race, and the outcome variable was three domains of healthcare access: insurance status, having any routine place for healthcare, and having any health visits in the past year. Chi-square tests and unadjusted and adjusted multivariate logistic regressions were conducted. The models were adjusted for age, education, income, and CKD stages and were weighted to account for the sampling strategy.

**Results:**

The sample size was 1864 CKD patients from three two-year cycles of NHANES datasets (2013–2014, 2015–2016, and 2017–2018). The final adjusted model found that Hispanic CKD patients were more likely to be uninsured (OR: 2.52, CI 1.66–3.83) and have no routine place for healthcare (OR: 1.68, CI 1.03–2.75) than White CKD patients, but did not have differences in healthcare visits in the past year.

**Conclusions:**

Hispanic CKD patients have limited healthcare access compared to White populations showing existing care access disparities experienced by them. Improved programs and policies are required to enhance kidney health among Hispanics and promote equity in CKD.

**Supplementary Information:**

The online version contains supplementary material available at 10.1186/s41043-024-00508-4.

## Background

In the year 2010, the International Society of Nephrology recognized chronic kidney disease (CKD) as a significant contributor to the global disease burden [1]. CKD has contributed to a significant mortality rate over the last few decades and became one of the fastest and most rising causes of death. By 2015, CKD has risen to become the 12th leading reason for death and 17th in global years of loss of life, constituting more than 1.1 million fatalities in the USA [2]. The management of end-stage CKD requires lifelong renal replacement therapies, the most expensive renal medicine, which requires an enormous healthcare budget [3].

In CKD, individuals experience a gradual decline in kidney function, primarily influenced by social determinants of health such as lower income and education levels, and poor housing conditions. Other risk factors include hazardous metabolic biomarkers, aging, obesity, diabetes, and hypertension, among others [4]. People of color, particularly Hispanics, show a higher prevalence of CKD risk factors, leading to increased incidence and prevalence of CKD within this group [5]. Currently, CKD is a public health concern in the USA, with 14% of the population diagnosed with the disease [6]. Hispanics have a similar CKD prevalence (13.7%) compared to the overall US population, but they are 1.3 times more likely to progress to kidney failure than Whites [6, 7]. This escalating CKD burden among Hispanics significantly contributes to the country’s overall CKD burden. Two previous major research initiatives, Chronic Renal Insufficiency Cohort (CRIC) study and Hispanic Chronic Renal Insufficiency Cohort (H-CRIC) study, found that Hispanics have significantly increased manifestation of kidney dysfunctions in terms of estimated glomerular filtration rate (eGFR), urine–protein ratio, left ventricular hypertrophy, and adverse metabolic biomarkers than Whites. These studies also revealed that Hispanics were less likely to achieve CKD management goals such as blood pressure control, blood pressure medication use, and cardiovascular disease prevention compared to Whites [8, 9]. This comprises a disproportionate disease burden within these populations, including higher rates of morbidities, extended hospitalizations, increased healthcare expenses, unemployment, disabilities, and poor quality of life experienced by Hispanics. Improved healthcare access among Hispanic CKD patients can reduce this burden, whereas lack of healthcare access may delay CKD diagnosis, accelerate disease progression, and increase disease severity [5, 10, 11]. Desai and colleagues discussed CKD disparities within the Hispanic population, emphasizing their lower likelihood of having regular healthcare providers, reporting previous contact with a nephrologist, and obtaining care from a nephrologist at least 12 months before starting dialysis treatment, compared to Whites. These factors are critical in the early stages for potentially improving kidney conditions and slowing disease progression [5, 12].

Despite representing the biggest ethnic minority group in the USA, in general, Hispanic populations exhibit limited healthcare access in the USA. A systematic review conducted in 2010 uncovered that Hispanic immigrants were at higher risk of not having healthcare access in the USA compared to Whites [13]. Pooled data from National Health Interview Surveys (NHIS) from 1999 to 2001 demonstrated that working-aged Hispanic adults, including Mexican Americans and Puerto Ricans, were less likely to have healthcare access than Whites [14]. NHIS data analysis from 1999 to 2002 also displayed that Hispanic young adults in the USA had a lower likelihood of having insurance and consistent sources of care than Whites [15]. In 2010, the Affordable Care Act (ACA) provided improved healthcare coverage for underserved groups in the USA [16]. This legislation was complemented by the launch of World Kidney Days in 2006; an annual health awareness campaign focused on highlighting healthcare access and health equity in CKD [17]. However, Hispanic CKD patients still struggle to achieve better kidney health outcomes [5, 18]. This persistent disparity underscores the need to understand healthcare access and utilization and existing care disparities for Hispanic CKD patients, especially for future policy-level changes. Previously published literature has addressed healthcare disparities experienced by Hispanic populations [13–15, 19, 20]. Multiple studies examining data from the pre-ACA periods have consistently reported healthcare access disparities among non-White CKD patients [21, 22]. Nonetheless, there exists a literature gap in terms of evaluating healthcare disparities experienced by Hispanic CKD patients, particularly during the current ACA era. Examining recent data can provide insights into whether progress has been achieved in the CKD care delivery system, especially for Hispanics. This study aims to evaluate the extent of racial disparities in healthcare access experienced by Hispanic CKD patients compared to Whites during the years 2013–2018. Our goal is to generate evidence and provide insights that will inform the development of future programs and policies that will promote kidney health equity in the USA.

## Methods

We used three two-year-cycle data sets from the National Health and Nutrition Examination Survey (NHANES). We examined healthcare access of Hispanic CKD patients in recent years, but before the onset of the COVID-19 pandemic. Therefore, we analyzed NHANES data from the years 2013–2018. The National Center for Health Statistics designed NHANES to assess the health and nutrition status of the US population and is conducted in English and Spanish languages. NHANES includes laboratory testing, physical examination, and interview datasets. NHANES participants are selected from different areas of the USA, ensuring diversity and representing a precise measure of health and nutrition indicators for various population groups [23]. NHANES datasets do not include personally identifiable information, accordingly, and consistent with the precedent set by other studies, institutional IRB approval was not sought for this study [24]. This study examined patient demographic data, relevant laboratory test results (urinary albumin and creatinine), and self-reported data (via a patient survey questionnaire) to investigate Hispanic CKD patients’ healthcare access disparities. Our population of interest included US Hispanic and White adults (aged ≥ 18) with CKD from stage-1 to stage-5. Firstly, all eligible respondents' eGFR (estimated glomerular filtration rate) was estimated using the CKD-EPI (Chronic Kidney Disease Epidemiology Collaboration) equation. Then, their CKD stages were identified following the 2012 KDIGO (Kidney Disease: Improving Global Outcomes) classification recommendations [25]. Anyone with the following criteria was excluded from the study: people not classified as CKD stage-1 to stage-5, people other than the White and Hispanic race, and people under the age of 18 years. To identify the CKD stages, firstly, eGFR (estimated glomerular filtration rate) was estimated for all adult (age ≥ 18) respondents across the three cycles. Then, the respondent's CKD stages were identified following the 2012 KDIGO classification. Therefore, we considered eGFR ≥ 90 with urinary albumin and creatinine ratio (ACR) ≥ 30 as CKD stage-1; eGFR 60–89 with ≥ ACR 30 as CKD stage-2; eGFR 30–59 as CKD stage-3; eGFR 15–29 as CKD stage-4; and eGFR less than 15 as CKD stage-5. We considered race the primary predictor variable and categorized race into two categories: non-Hispanic Whites and Hispanics. Other racial groups were excluded from the analysis. Three indicators of self-reported healthcare access from the NHANES interview dataset were examined as response variables: insurance status, the routine place for healthcare, and healthcare visits in the past one year. Being uninsured, having no routine place for healthcare, or having no healthcare visit in the past one year were considered limited healthcare access. Covariates for the analysis included age (≤ 24 years, 25–44, 45–64, 65 or above), sex (male and female), CKD awareness (ever told about weak or failing kidneys, excluding kidney stones, bladder infections, or incontinence), CKD stages (stage-1 to stage-5), household income (less than $75,000/year and more than and equal to $75,000/year, the US median household income), and education (high school or less and more than high school) [26]. Chi-square tests and multivariable logistic regressions were conducted in Statistical Analysis System (SAS). Missing values were less than 10% for the analyzed variables. We used data weighting techniques with the NHANES dataset to ensure that our analyses accurately represent the US population. By applying sample weights to each participant’s data during our analysis, we accounted for the survey’s complex sampling design, including oversampling, non-response, and post-stratification adjustments. This weighting approach enhances the external validity of our findings, offering a more accurate reflection of the health characteristics of the US population. Detailed information on the weighting methodology is available in the NHANES documentation. NHANES sample weights represent the U.S. Civilian Noninstitutionalized Census population [23].

## Results

**Fig. 1:**
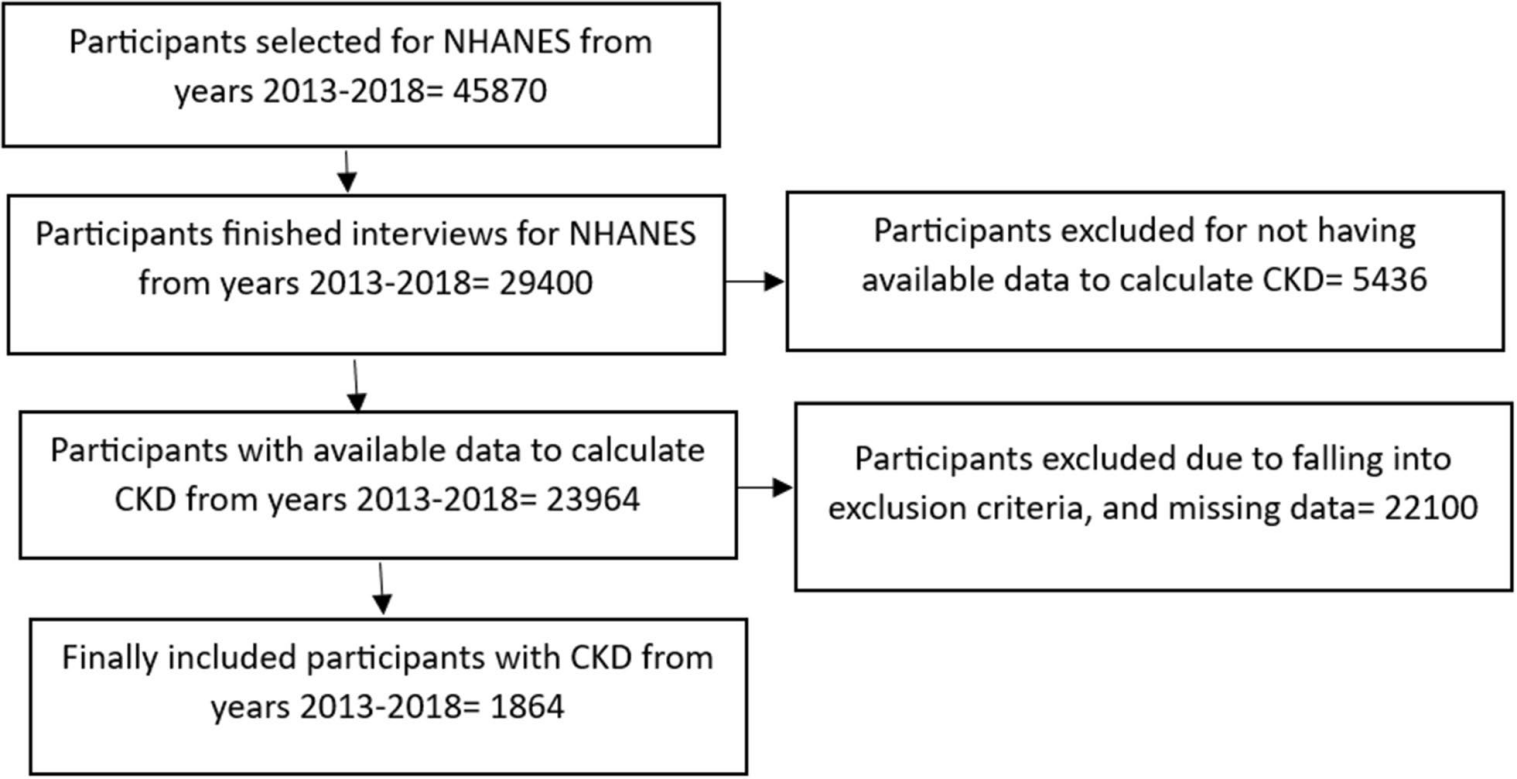
Study flowchart

**Fig. 2:**
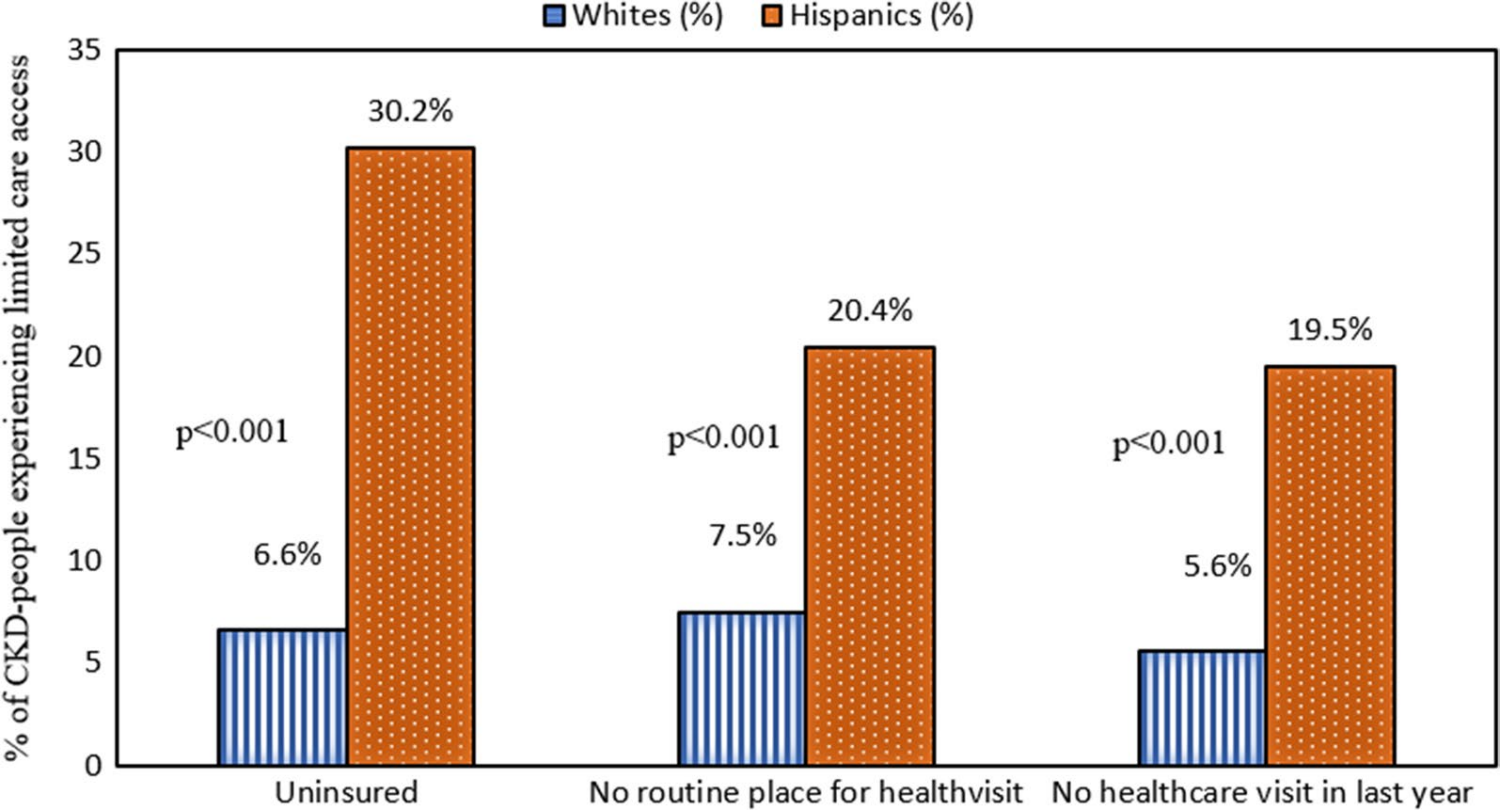
Disparities in healthcare access in percentage (%)

A total of 45,870 participants were selected to participate in NHANES study for the three cycles (Fig. 1). Ultimately, 1864 CKD patients from the dataset were included in our analysis, among which 84.5% were Whites, and 15.5% were Hispanics. We found that Hispanic CKD patients had less education and income than White CKD patients (Table 1). In 2013–2018, a significantly greater percentage of Hispanic CKD patients had less than high school level education compared to that of Whites (*p* < 0.0001). Similarly, a significantly higher percentage of Hispanic CKD patients had income < 75 K than Whites (*p* = 0.002). Age and CKD stages distributions among Hispanic CKD patients were significantly different compared to the White CKD patients (< 0.0001). Hispanic CKD patients tended to be younger than White patients. Specifically, among Hispanics, there was a higher percentage of individuals aged 45–64 (33.2%), followed by age ranges: 25–44, ≥ 65, and ≤ 24, respectively. In contrast, among Whites, most CKD patients were aged more or equal to age 65 (58.2%), followed by age groups: 45–64, 25–44, and ≤ 24. Among Hispanics, the highest percentages of CKD patients were found to be in the CKD stage-1 category (57%), whereas, among Whites, the highest percentages of CKD patients belonged to the CKD stage-3 category (51.6%). Whites and Hispanics did not have a significantly different awareness of their CKD status. Sex was not associated with race among CKD patients. Figure 2 showed that compared to Whites, a higher percentage of Hispanics were uninsured (30.2% vs. 6.6%), had no routine place to visit healthcare (20.4% vs. 7.5%), and had no healthcare visit in the past one year (19.5% vs. 5.6%) (*p* < 0.001).

**Table 1:** The findings of Chi-square tests of the demographic variables by race (weighted), *n* = 1864

	Whites (weighted values)	Whites (%)	Hispanics (weighted values)	Hispanics (%)	*p* value
Weighted sample size	22,763,252	84.5	4,161,779	15.5	< 0.0001
Sex					0.68
Female	13,099,718	57.5	2,342,614	56.3	
Male	9,663,534	42.4	1,819,166	43.7	
Age					< 0.0001
≤ 24	811,647	3.6	251,255	6	
25–44	2,549,468	11.2	1,336,418	32.1	
45–64	6,149,249	27	1,383,527	33.2	
≥ 65	13,252,887	58.2	1,190,579	28.6	
Education					< 0.0001
High school or less	8,920,097	39.2	280,427	67.5	
More than high school	13,826,468	60.8	135,111	32.5	
Annual household income					< 0.0001
≥ 75 K	800,742	36.2	687,686	18.8	
< 75 K	14,087,371	63.8	2,976,173	81.2	
Awareness of CKD status					0.86
Aware	2,411,105	10.7	452,802	10.9	
Unaware	20,217,434	89.3	3,699,568	89.1	
CKD stages					< 0.0001
Stage-1	5,677,111	24.9	237,046	57	
Stage-2	4,649,478	20.4	708,100	17	
Stage-3	11,749,861	51.6	901,093	21.7	
Stage-4	512,842	2.3	132,608	3.2	
Stage-5	173,960	0.8	49,517	1.2

We used five logistic regression models to adjust for confounding. The dependent variables for these models were insurance status (uninsured), the absence of a routine place for healthcare, and the absence of a healthcare visit in the past year. The main predictor in these models was race, specifically comparing Hispanics to Whites. The detailed findings are attached in the additional materials (see additional material). All the models for the uninsured status and no routine place for healthcare visits found that Hispanics are more likely to have limited healthcare access (Additional file 1: Table S1 and Table S2). Final models in Table 2 found that Hispanic CKD patients have 2.52 (CI 1.66–3.83) times the odds of being uninsured and 1.68 (CI 1.03–2.75) times the odds of having no routine place for healthcare compared to White CKD patients. Though Hispanics tended to have lower odds of a healthcare visit in the past year, and this difference was significant in the unadjusted model, the relationship between the variables attenuated when the CKD stage was considered in the model and confidence intervals for the full model slightly overlapped the null (Table 3). Table 3 displays the results of logistic regression models examining the correlation between race (Hispanics versus Whites) and the likelihood of not having visited a healthcare facility in the past year. The crude model (Model 1) indicates that Hispanics are 4.10 times more likely to lack a healthcare visit, showing an unadjusted association. After adjusting for age (Model 2), the odds ratio decreases to 2.44, suggesting the influence of age-related factors. Further adjustments for education (Model 3) and income (Model 4) result in odds ratios of 1.94 and 1.72, respectively, highlighting the impact of socioeconomic factors. The final model (Model 5), adjusted for age, education, income, and CKD stages, shows an odds ratio of 1.61. This comprehensive model demonstrates a persistent yet reduced association between race and the absence of a healthcare visit, emphasizing the importance of considering demographic, socioeconomic, and health-related factors in understanding healthcare access disparities. Additionally, the odds ratios for various age categories, education levels, income, and CKD stages offer valuable insights into the complex factors contributing to this relationship. These results indicated a racial disparity in healthcare access between Hispanic and White CKD patients.

**Table 2:** Association of race and limited healthcare access in terms of logistic regressions (weighted)

Hispanics versus Whites	Unadjusted OR (95% CI)	Adjusted OR (95% CI)*
Uninsured	6.08 (4.19–8.82)	2.52 (1.66–3.83)
No routine place for healthcare	3.17 (2.06–4.87)	1.68 (1.03–2.75)
No healthcare visit in the past 1 year	4.10 (2.73–6.16)	1.61 (0.97–2.66)

*OR* odds ratio, *CI* confidence interval

*The final model is adjusted by age, education, income, and CKD stage

**Table 3:** Logistic regression models for race and no healthcare visit in past year

	Model 1 OR (95% CI)	Model 2 OR (95% CI)	Model 3 OR (95% CI)	Model 4 OR (95% CI)	Model 5 OR (95% CI)
Hispanics versus Whites (reference)	4.10 (2.73–6.16)	2.44 (1.54–3.87)	1.94 (1.19–3.18)	1.72 (1.08–2.75)	1.61 (0.97–2.66)
Age 25–44 versus ≤ 24 (reference)		0.91 (0.39–2.12)	0.97 (0.40–2.36)	0.90 (0.38–2.14)	0.98 (0.38–2.51)
Age 45–64 versus ≤ 24 (reference)		0.44 (0.20–0.95)	0.46 (0.2–1.06)	0.46 (0.2–1.07)	0.63 (0.23–1.71)
Age ≥ 65 versus ≤ 24 (reference)		0.07 (0.03–0.15)	0.069 (0.03–0.15)	0.06 (0.02–0.14)	0.14 (0.04–0.45)
Education: more than high school vs high school or less (reference)			0.41 (0.23–0.72)	0.49 (0.27–0.89)	0.49 (0.27–0.92)
Income < 75 K versus ≥ 75 K (reference)				1.49 (0.76–2.93)	1.46 (0.75–2.84)
CKD stage-1 versus advanced CKD (reference)					6.03 (1.42–25.63)
CKD stage-2 versus advanced CKD (reference)					4.96 (1.07–22.98)
CKD stage-3 versus advanced CKD (reference)					1.77 (0.38–8.23)

Model 1: crude; model 2: adjusted by age; model 3: adjusted by age and education; model 4: adjusted by age, education, and income; model 5 or final model: adjusted by age, education, income, and CKD stages. We have added advanced CKD (combination of stages 4 and 5) as reference group of CKD stages to ensure sufficient power in each category)

## Discussion

We found that compared to non-Hispanic Whites, Hispanic CKD patients tended to be less educated, had lower household incomes, were younger, and had earlier stage of the disease. Recognizing these demographic and socioeconomic differences is key to understanding the existing health disparities, planning effective public health programs and improving policies that advance equity in kidney health. The link between lower socioeconomic status and unfavorable dialysis outcomes among racially underrepresented groups has been discussed previously by other researchers [27]. Considering the disadvantaged socioeconomic circumstances of our Hispanic participants, ensuring they receive adequate healthcare is critical to mitigate the risk of future dialysis complications.

In addition, our study found that Hispanic CKD patients had limited healthcare access compared to Whites, confirming the presence of kidney care disparities in the USA. Our unadjusted and adjusted models found that Hispanic CKD patients were less likely to have insurance coverage or have any routine place for health-related visits. Our findings are consistent with the findings of Agrawal and his colleagues, who reported that Hispanic CKD patients were less likely to be insured (48.1%) than their White counterparts (90.9%). Agrawal et al. analyzed patient participation data from the National Kidney Foundation's health screening program, Kidney Early Evaluation Program (KEEP), from 2000 to 2010. Our analysis of the post-KEEP NHANES data confirms the presence of continuing disparities in kidney care access, indicating a need for ongoing strategic interventions across the nation [22]. In 2010, the ACA was adopted to improve the healthcare access of the underserved US population. While overall insurance coverage increased in the post-ACA period, the uninsured rate among Hispanics remained high [19]. In the post-ACA implementation era, healthcare access for Hispanics improved more than non-Hispanic Whites. Hispanics were less likely to forgo physician visits compared to Whites [20]. However, in recent years, the ACA's achievements seem to have moderated. Even if patients have insurance coverage, language barriers, mistrust toward the healthcare system, and lack of cultural competencies by healthcare workers detract Hispanic CKD patients from receiving regular care [5, 19, 28]. Our findings also align with the findings of a Pew research centers’ report which found that Hispanics were less likely to have a regular healthcare provider. According to their report, more than double the proportion of Hispanics had no regular place for health-related visits when compared to Whites [5, 29]. Additionally, research by the Centers for Disease Control and Prevention (CDC) mentioned that Hispanic adults were less likely to have regular healthcare providers than non-Hispanic Whites and Blacks [29, 30].

Our final adjusted logistic regression model (model 5) demonstrated an association between CKD patients being Hispanics and the outcome variable of having a healthcare visit in the past year, but this association was not statistically significant. However, other models for these two variables found statistically significant associations. It seems that the CKD stage had an impact on the relationship between these variables (Table 3). Healthcare utilization of CKD patients increases in the advanced stages of CKD compared to the early stages. In stage-1 and stage-2, CKD often remains asymptomatic, which might be a reason why early-stage CKD patients have a lower tendency to visit health centers [31]. Many people remain in the early stages for months or years without symptoms [31]. By enhancing healthcare utilization during this phase, individuals would have the opportunity to receive assistance from healthcare professionals early, enabling them to effectively manage CKD and potentially halt its progression. Conversely, advanced staged CKD patients, especially in stage-4 and 5, require frequent hospital visits, dialysis center visits, and specialized medical attention due to their deteriorating health [22]. However, previously published literature expressed that Hispanics had limited healthcare utilization behaviors compared to Whites [22, 32]. More Hispanic KEEP participants also reported having extremely or moderately difficult healthcare access than non-Hispanic White or Black CKD patients [22].

Multiple intersecting factors may influence healthcare access disparities. Language barriers, acculturation, miscommunications between providers and patients, lack of support, and distinguished cultural values inevitably affect Hispanics’ access to the formal care system [11, 33]. While seeking health, many Hispanic CKD patients prefer religious, spiritual, and cultural remedies over standard medical care [34]. Additionally, staying in the USA as undocumented immigrants, especially for many Hispanics with CKD, makes them ineligible for Medicare or many other insurances, which decreases their likelihood of receiving any systematic care before dialysis and late diagnosis of CKD [35]. The delayed identification of CKD exacerbates the need for costly emergency dialysis, placing a significant economic strain on the nation [11]. Undocumented immigrants even lack adequate coverage for Medicaid and kidney transplantation. Notably, individuals more than 65 years old and patients with CKD and dialysis are largely covered by Medicare in the USA [36]. Medicare covers around 80% of the dialysis expenses and cost of some medications after kidney transplantation and generally starts covering from the fourth month of dialysis initiation (the first month in terms of home dialysis)[37, 38]. Even if individuals have Medicare or other insurance, they might be in need of supplemental plans to cover doctors’ visits, medications, diagnostic tests, nutritional counseling, and other supportive services [37, 39]. Patient Protection and ACA expanded private insurance marketplaces, enhancing enrollment periods, and offering affordable coverage to patients with preexisting conditions. ACA’s Medicaid expansion in many states enabled many low-income individuals, including those with Hispanic CKD or dialysis patients, to have insurance coverage and ensure better care access. However, Hispanic CKD patients in non-expanding states are still ineligible for Medicaid or subsidies, limiting their healthcare access [38]. The passage of ACA and the expansion of Medicare also improved healthcare utilization and preemptive listings for transplantation for people with color [16, 20]. In 2019, The Advancing American Kidney Health (AAKH) initiative was introduced with the aspiration of transforming kidney care nationwide. However, the AAKH initiative did not explicitly address health equity concerns. Recently, an executive order was announced on Advancing Racial Equity and its goal is to promote kidney health and healthcare equity for historically underserved populations [40]. Future research is needed to understand how these recent policies have influenced racial disparities in kidney care.

Improved programs and policies prioritizing the social determinants of health could significantly improve kidney health among Hispanics and advance equity in CKD [40, 41]. We recommend developing and implementing outreach programs that enhance engagement and education about CKD, resonating with Hispanic cultural values and practices. Expanding training and reimbursement for Community Health Workers could be effective in supporting Hispanic communities with prevention, early detection, and assistance in many ways to slow disease progression. Other recommendations for enhancing the quality of care and health outcomes for Hispanics include improving interpretation services in health clinics and increasing diversity and representation within healthcare workforces. Additionally, more restrictions on Medicaid coverage should be removed, and access to affordable marketplace or employer coverage should be improved. Besides increasing assistance programs for medications, dialysis, transportation to medical appointments, and caregiver support, allocating more funding in equity research in the realm of kidney space is recommended [42].

This study offered valuable insights into existing healthcare disparities experienced by Hispanic CKD patients in the post-ACA period compared to Whites. Following Aday and Andersen’s (1975) definition of healthcare access, we selected variables that define both actual and potential entries of individuals into healthcare [43]. Among all our response variables, healthcare visits in the past one year described CKD patients' actual entries of healthcare, whereas insurance status and having any routine place for healthcare explained patients' potential entry into healthcare. This study’s findings should be interpreted considering a few limitations. Most study variables were obtained through a self-reported questionnaire. NHANES participants might struggle to accurately recall past experiences or events, leading to a collection of partial information [23]. Additionally, cross-sectional analysis does not infer causality. NHANES uses limited instruments to measure healthcare access. Therefore, many perspectives to measure healthcare access, such as providers' and specialized care availabilities, transportation accessibilities, cultural, social, and language barriers, and discrimination and biases in the system, have yet to be explored. Additionally, since NHANES does not include geographic locations or state parameters, we were unable to compare the data of participants residing in states with Medicaid expansion versus those without. Future studies are needed to delve into these aspects to better measure healthcare access among Hispanic CKD patients.

## Conclusions

This study highlights a healthcare access gap among US individuals with CKD. Although recent policy changes have been implemented, disparities in healthcare access continue, disproportionately affecting Hispanic CKD patients. This underscores the need for improved programs and policies to advance health equity in CKD.

### Supplementary Information

**Additional file 1: Table S1**. Adjusted and unadjusted models for logistic regressions between race and being uninsured, **Table S2**. Adjusted and unadjusted models for logistic regressions between race and having any routine place for healthcare.

## Data Availability

The datasets are available online in CDC’s website (National Health and Nutrition Examination Survey. Centers for Disease control and Prevention. Available from: https://wwwn.cdc.gov/nchs/nhanes/Default.aspx). For further guidance, please feel free to contact the corresponding author.
