# Supplementary material for: Disparities in healthcare access experienced by Hispanic chronic kidney disease patients: a cross-sectional analysis
Source: J Health Popul Nutr. 2024 Jan 31;43:18. doi: 10.1186/s41043-024-00508-4 (PMC10832131; doi:10.1186/s41043-024-00508-4)
Supplement: Supplementary file 1 — Additional file 1: Table S1. Adjusted and unadjusted models for logistic regressions between race and being uninsured, Table S2. Adjusted and unadjusted models for logistic regressions between race and having any routine place for healthcare. [file 41043_2024_508_MOESM1_ESM.docx]

**Title: Disparities in Healthcare Access Experienced by Hispanic Chronic Kidney Disease Patients: A Cross Sectional Analysis**

**Supplementary materials:**

Supplementary materials in Microsoft Word document. This includes three tables (Table S1. Adjusted and unadjusted models for logistic regressions between race and being uninsured, Table S2. Adjusted and unadjusted models for logistic regressions between race and having any routine place for healthcare, Table S3**.** Adjusted and unadjusted models for logistic regressions between race and no healthcare visit in the past one year)

**Table S1.** Unadjusted and adjusted models for logistic regressions between race and being uninsured.

|  | **Model 1**  **OR**  **(95% CI)** | **Model 2**  **OR**  **(95% CI)** | **Model 3**  **OR**  **(95% CI)** | **Model 4**  **OR**  **(95% CI)** | **Model 5**  **OR**  **(95% CI)** |
| --- | --- | --- | --- | --- | --- |
| **Hispanics vs**  **Whites (reference)** | 6.08  (4.19-8.82) | 4.13  (2.88-5.91) | 3.34  (2.34-4.77) | 2.60  (1.76-3.85) | 2.52  (1.66-3.83) |
| **Age 25-44 vs**  **≤ 24 (reference)** |  | 0.58  (0.30-1.12) | 0.61  (0.31-1.17) | 0.52  (0.28-.98) | 0.56  (0.29-1.07) |
| **Age 45-64 vs**  **≤ 24 (reference)** |  | 0.47  (0.26-0.86) | 0.49  (0.27-0.91) | 0.41  (0.22-0.74) | 0.50  (0.25-.99) |
| **Age ≥ 65 vs**  **≤ 24 (reference)** |  | 0.06  (0.03-0.14) | 0.06  (0.03-0.14) | 0.05  (0.02-0.11) | 0.08  (0.03-0.21) |
| **Education: more than high school vs high school or less (reference)** |  |  | 0.39  (0.24-0.62) | 0.45  (0.28-0.82) | 0.49  (0.28-0.83) |
| **Income < 75K vs**  **≥75K (reference)** |  |  |  | 2.54  (1.34-4.82) | 2.55  (1.34-4.86) |
| **CKD stage-1 vs**  **advanced CKD (reference)** |  |  |  |  | 4.18  (1.13-15.52) |
| **CKD stage-2 vs**  **advanced CKD (reference)** |  |  |  |  | 3.27  (0.77-13.83) |
| **CKD stage-3 vs**  **advanced CKD (reference)** |  |  |  |  | 2.41  (0.57-10.20) |

model-1: crude; model-2: adjusted by age; model-3: adjusted by age and education; model-4: adjusted by age, education, and income; model-5 or final model: adjusted by age, education, income, and CKD stages

Here, we have added advanced CKD (combination of stage-4 and stage-5) as reference group of CKD stages to ensure sufficient power in each category.

**Table S2.** Unadjusted and adjusted models for logistic regressions between race and having any routine place for healthcare.

|  | **Model 1**  **OR**  **(95% CI)** | **Model 2**  **OR**  **(95% CI)** | **Model 3**  **OR**  **(95% CI)** | **Model 4**  **OR**  **(95% CI)** | **Model 5**  **OR**  **(95% CI)** |
| --- | --- | --- | --- | --- | --- |
| **Hispanics vs**  **Whites (reference)** | 3.17  (2.06-4.87) | 2.00  (1.28-3.13) | 1.93  (1.26-2.98) | 1.69  (1.05-2.72) | 1.68  (1.03-2.75) |
| **Age 25-44 vs**  **≤ 24 (reference)** |  | 0.40  (0.19-0.82) | 0.40  (0.2-0.83) | 0.38  (0.19-0.78) | 0.40  (0.19-0.82) |
| **Age 45-64 vs**  **≤ 24 (reference)** |  | 0.27  (0.13-0.59) | 0.30  (0.13-0.60) | 0.25  (0.11-0.56) | 0.29  (0.13-0.64) |
| **Age ≥ 65 vs**  **≤ 24 (reference)** |  | 0.04  (0.02-0.10) | 0.04  (0.01-0.11) | 0.04  (0.02-0.1) | 0.05  (0.02-0.15) |
| **Education: more than high school vs high school or less (reference)** |  |  | 0.90  (0.52-1.49) | 0.95  (0.55-1.65) | 0.95  (0.55-1.67) |
| **Income < 75K vs**  **≥75K (reference)** |  |  |  | 1.25  (0.65-2.39) | 1.24  (0.64-2.42) |
| **CKD stage-1 vs**  **advanced CKD (reference)** |  |  |  |  | 5.97  (1.18-30.16) |
| **CKD stage-2 vs**  **advanced CKD (reference)** |  |  |  |  | 6.62  (1.03-30.7) |
| **CKD stage-3 vs**  **advanced CKD (reference)** |  |  |  |  | 4.34  (0.85-22.18) |

model-1: crude; model-2: adjusted by age; model-3: adjusted by age and education; model-4: adjusted by age, education, and income; model-5 or final model: adjusted by age, education, income, and CKD stages

Here, we have added advanced CKD (combination of stage-4 and stage-5) as reference group of CKD stages to ensure sufficient power in each category.
